# Immunology of Cryptococcal Infections: Developing a Rational Approach to Patient Therapy

**DOI:** 10.3389/fimmu.2018.00651

**Published:** 2018-04-04

**Authors:** Waleed Elsegeiny, Kieren A. Marr, Peter R. Williamson

**Affiliations:** ^1^Laboratory of Clinical Immunology and Microbiology (LCIM), National Institute of Allergy and Infectious Diseases (NIAID), National Institutes of Health (NIH), Bethesda, MD, United States; ^2^Johns Hopkins University, Baltimore, MD, United States

**Keywords:** *Cryptococcus*, cryptococcal, meningoencephalitis, meningitis, neurology, infection, fungus

## Abstract

Cryptococcal meningoencephalitis is responsible for upwards of 15% of HIV-related deaths worldwide and is currently the most common cause of non-viral meningitis in the US, affecting both previously healthy and people with immune suppression caused by cancer chemotherapy, transplantation, and biologic therapies. Despite a continued 30–50% attributable mortality, recommended therapeutic strategies have remained largely unchanged since the 1950s. Recent murine models and human studies examining the role of the immune system in both susceptibility to the infection as well as host damage have begun to influence patient care decisions. The Damage Framework Response, originally proposed in 1999, was recently used to discuss dichotomous etiologies of host damage in cryptococcal disease. These include patients suffering microbiological damage with low host immunity (especially those immunosuppressed with HIV) and those having low (live) microbiological burden but high immune-mediated damage (HIV-related immune reconstitution syndrome and non-HIV-related postinfectious inflammatory response syndrome). Cryptococcal disease in previously healthy hosts, albeit rare, has been known for a long time. Immunophenotyping and dendritic cell-T cell signaling studies on cerebral spinal fluid of these rare patients reveal immune capacity for recognition and T-cell activation pathways including increased levels of HLA-DR and CD56. However, despite effective T-cell signals, brain biopsy and autopsy specimens demonstrated an M2 alternative macrophage polarization and poor phagocytosis of fungal cells. These studies expand the paradigm for cryptococcal disease susceptibility to include a prominent role for immune-mediated damage and suggest a need for careful individual consideration of immune activation during therapy of cryptococcal disease in diverse hosts.

## Introduction

*Cryptococcus* is an opportunistic fungus, which most frequently presents as a pulmonary infection or meningoencephalitis. Cryptococcosis has a high impact on immunocompromised populations such as patients with HIV-AIDS and a wide array of non-HIV patients including those with hematopoietic malignancies, autoimmune diseases, or genetic immunodeficiency syndromes, as well as patients receiving immunosuppressive cancer-therapies, undergoing transplant conditioning, in combination with age-related immunosenescence (1–5). HIV-related cryptococcal meningitis (CM) is one the most common causes of adult meningitis with an estimated 223,100 cases and 181,100 deaths in 2014, globally (6, 7). In countries with access to optimal medical care, non-HIV CM accounts for at least 25% of all CM-related hospitalization and deaths and is currently the leading cause of non-viral meningitis in the U.S. (2, 8–10). Interestingly, there are rare reports that as much as 30% of non-HIV patients with cryptococcal infection were previously healthy with no known underlying condition (11).

*Cryptococcus* is a basidiomycete yeast with over 30 known species; however, the majority of human infections are caused by either *Cryptococcus neoformans* and *Cryptococcus gattii*. *C. neoformans* is the main source of infections in CM patients with CD4+ T-cell deficiency while *C. gattii* is a predominant species in the previously healthy (12, 13). *C. neoformans* and *C. gattii* both can both be found in the vicinity of a variety of trees, and *C. neoformans* can also be found in soils and bird feces (14). Although the life-cycle of *Cryptococcus* is not dependent on an animal host, *C. neoformans* has the potential to infect a wide range of warm- and cold-blooded species (15). Cryptococci are considered sapronotic due to their ability to cause an opportunistic infection without coevolution of a host–parasite virulence (16) although molecular optimization of virulence has been noted in environmental strains after mammalian residence (17). Key to infecting such a wide-ranging host population is its adaptation to environmental conditions and defenses against innate plant defenses as well as phagocytic predators such as parasites and insects (18–21).

## Cryptococcal Infection

Cryptococcal infection is believed to be transmitted by inhalation of infective particles such as yeast cells and/or spores from an environmental source. It is believed that humans encounter the organism early in life, evident by the gradual increase of cryptococcal-specific antibodies in humans with age (22) and isolation of infective strain types from the country of origin in immigrant patients later presenting with HIV/AIDS-related CM (23). As most immunocompetent humans are asymptomatic and resolve the infection, there are limited observations as to the mechanism in which infection is cleared. *Cryptococcus* spp. has a unique repertoire of immune reactivity from other fungi because of distinguishing attributes such as a large polysaccharide capsule that limits exposure to immune dominant carbohydrate epitopes (20), immunomodulatory enzymes such as a phenol oxidizing and cytokine-inducing laccase (24), and a robust tolerance to low nutrient conditions such as within brain tissues (25, 26).

## Innate Recognition

Although no single pattern recognition receptor (PRR) has been shown to be required for binding of *Cryptococcus*, it is hypothesized that alveolar macrophages recognize *Cryptococcus* and initialize the immune response through multiple receptors such as Dectin-1, Mincle, mannose receptor, CD14, and toll-like receptors (TLRs) (Figure 1.1) (27–30). The role of AMs and phagocytosis is believed to be critical during early infection, as observed *in vivo* imaging studies and animal models demonstrating enhanced susceptibility after AM depletion (31, 32). The complexity of how PRRs impact the immune response to cryptococcal infection is still being studied as there may be redundancy among them. For example, TLRs 2, 4, and 9 individually appear to play only a minor role, although the use of agonists *in vitro* enhances the proinflammatory responses by microglial cells to *Cryptococcus* (33). NOD-like receptor family pyrin domain containing 3 (NLRP3) is another cryptococcal recognizing PRR and has been shown in mice to be involved with leukocyte infiltration in the lung during infection (34). Interestingly, *in vitro*, NLRP3 activation appears to be inhibited by capsulated cryptococcal cells further suggesting that PRRs may have a more important role during early infection (35). Some PRRs have been linked to specific immune responses such as scavenger receptors B1 and SR-B3, which appear to be important for the induction of IL-1β (36). Macrophage receptor with collagenous structure (MARCO) is also a scavenger receptor which plays a role in cellular recruitment to the lung, cytokine production, and pathogen uptake by mononuclear phagocytes during early stages of cryptococcal infection (37). However, during the adaptive phase of infection, mice deficient in MARCO have improved fungal clearance which is marker by a type-I skewed immune response. Although MARCO plays an important role initially during infection, it is believed that *Cryptococcus* is capable of exploiting MARCO to polarize toward a non-protective immune response (38). There are other recognition receptors such as scavenger receptor A (SR-A) that are also associated with poorer response to *Cryptococcus*. SR-A-deficient mice show enhanced fungal clearance, which was correlated with decreased production of IL-4 and IL-13 (39). This may be linked to the unique cell wall composition of *C. neoformans* compared to other fungi, which contains high levels of acetylated chitin and deacetylated chitosan polymers (40). Furthermore, the binding of PRRs will become hindered as the infectious propagule starts forming the polysaccharide capsule, thus, it is believed that the successful phagocytosis of *Cryptococcus* also requires antibody- or complement-type opsonins (41, 42).

**Figure 1:**
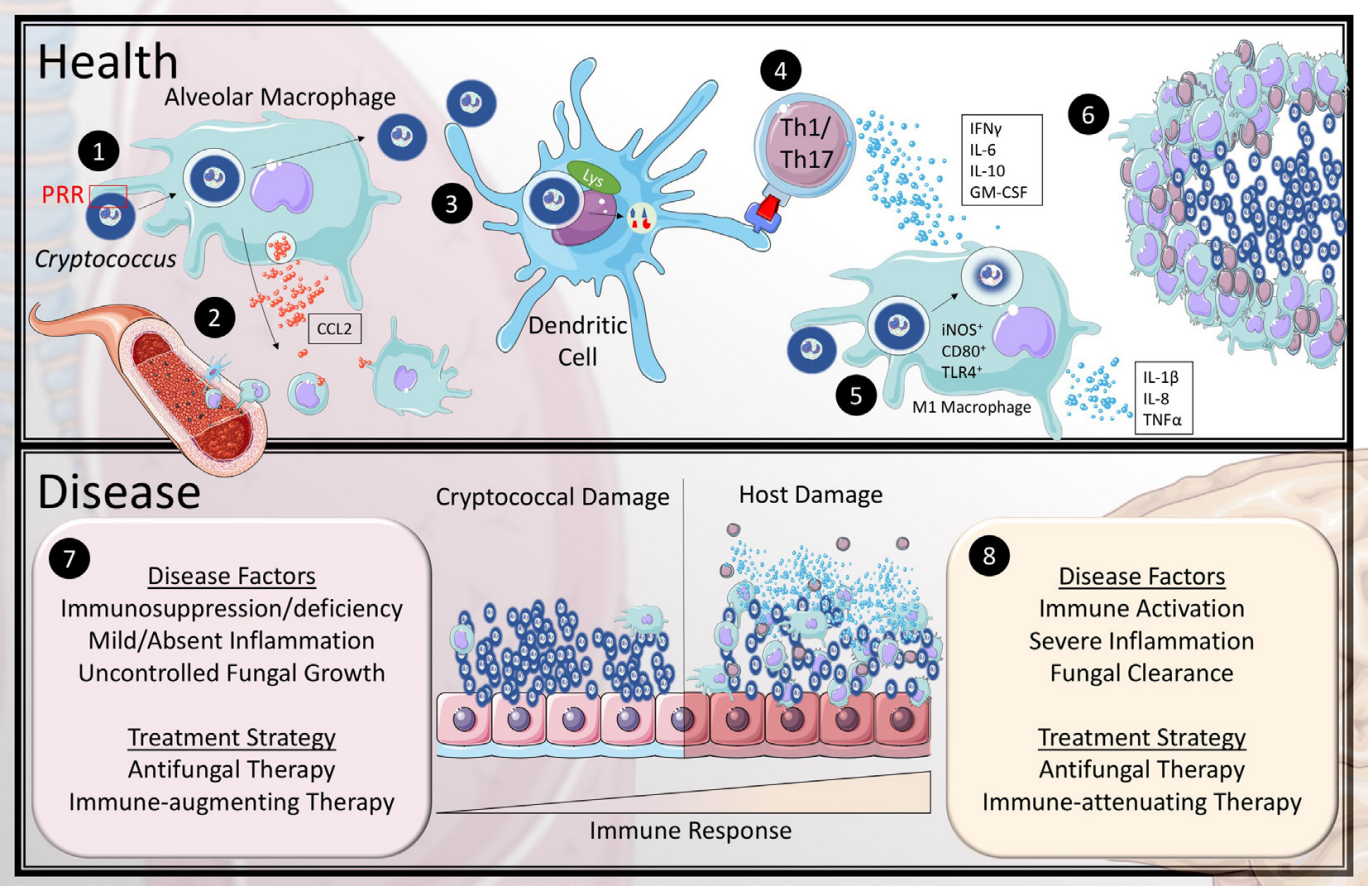
Current model of the immune response to *Cryptococcus* in health and disease. In health (1) inhaled cryptococcal spores are recognized by alveolar macrophages through pattern recognition receptors (PRR). (2) This stimulates the macrophages to release CCL2 to recruit monocytes and dendritic cells (DCs) to the lung. (3) Recruited DCs are capable of breaking down cryptococcal lifeforms and present antigen to CD4+ T-cells. (4) Activated T helper 1 and Th17 T-cells secrete IFNγ, IL-6, IL-10, and granulocyte-macrophage colony stimulating factor (GM-CSF) to recruit and differentiate classical (M1) macrophages. (5) The exact mechanism of fungicidal activity by M1 macrophages against *Cryptococcus* is still unclear in humans; however, they are known to upregulate iNOS, CD80, and TLR4 as well as produce IL-1β, IL-8, and TNFα. (6) The cytokine and chemokine mileu organize the leukocytes to encapsulate and eliminate cryptococcal organisms within granulomas. The most common association with cryptococcal disease (7) is the absence or dysfunction of at least one aspect of the healthy immune response, which leads to uncontrolled fungal growth. (8) However, some patients develop a skewed hyper-immune response to the pathogen that causes inflammatory damage to the host tissue even with fungal clearance.

## Initiation of the Immune Response

Although recognition and phagocytosis are important in induction of the immune response to *Cryptococcus*, the processes and pathways involved in breaking down and clearing pathogens and their antigens are also critical steps to mounting an optimal immune response. One clinical study observed that *in vitro* macrophage phagocytosis was directly correlated with clinical outcome (43). This suggests that other factors including phagocyte polarization and lysosomal activity may also need to be regulated for successful clearance.

Unlike other intracellular pathogens such as *Mycobacterium tuberculosis*, cryptococci do not interfere with phagosome formation or maturation; however, they are capable of surviving within vesicles or escaping by phagosome permeabilization or vesicular release (44–47). *C. neoformans* is thus capable of using macrophages as a host for immune evasion, and can escape through expulsion, lysis, or rupture due to excessive intracellular proliferation (47, 48). Alveolar macrophages are required; however, to recruit monocytes and dendritic cells (DCs) primarily through the production of macrophage chemotactic protein 1 (MCP1). In rats, MCP1 (also known as CCL2) and its receptor, CCR2, are essential for the recruitment of DCs, formation of granulomas, antigen presentation, and T-cell responses (Figure 1.2) (49, 50). Granulomas are a sign for control of infections and are composed of macrophages and giant multinucleated cells that contain cryptococcal cells, as well as CD4+ T-cells. These granulomas encompass the fungi and often resolve without additional medical assistance, but treatment with antifungal therapy or surgical removal of the lesions may expedite recovery. It has also been suggested that cryptococci may also be able to latently persist within granulomas and macrophages without degradation (51, 52). In patients with HIV-related pulmonary cryptococcosis, multinucleated giant cells are still present; however, the cryptococci are mainly extracellular and propagate within alveolar spaces (53). DCs are considered the primary antigen-presenting cell (APC) in the context of cryptococcal infection and have an advantage over macrophages in stimulating T-cell proliferation (54). Recruited DCs phagocytose cryptococcal bodies, which then are passaged through lysosomes to be degraded by both oxidative and non-oxidative mechanisms (Figure 1.3) (54, 55). For example, cathepsin-B has a non-enzymatic role to fracture the cell wall through osmotic lysis. Degraded components are then loaded onto major histocompatibility complex class II to initiate the adaptive immune response through CD4+ T-cell stimulation (Figure 1.4) (56). Eosinophils from a rat model of cryptococcosis have also been demonstrated to have the ability to phagocytose cryptococci and prime of CD4+ and CD8+ T-cells, *in vitro* (57) although their role in human infections is less clear. Their ability to function as APCs is associated with a decrease in nitric oxide and hydrogen peroxide production, followed by migration to the lymphatic system (58). However, their involvement in priming the adaptive immune response is associated with increased fungal burden and lung pathology by skewing immunity toward a type-II response (59, 60).

## The Adaptive Immune Response

Involvement of the adaptive immune compartment is critical for control of a cryptococcal infection; however, it may also have a detrimental affect depending on the type of response. In draining lymphoid tissues, APCs carrying cryptococcal antigens stimulate several types of lymphocytes including CD4+ T cells, CD8+ T cells, and natural killer T cells. Once activated, CD4+ T-cells can further differentiate into unique effector subsets with distinctive cytokine profiles including: T helper 1 (Th1), Th2, and Th17 cells. Cxcr5+ T follicular helper cells are also induced and primarily function to stimulate B cell maturation and antibody production, as well as activate inflammatory macrophages (61, 62). Th1 and Th17 cells are recognized by their production of IFNγ and IL-17, respectively, and both help mediate the resolution of cryptococcal infection. On the other hand, Th2 cells, which are described as producers of IL-4, IL-5, and IL-13, are associated with more of a detrimental outcome such as increases in inflammation, worsened pathology, and increased risk of dissemination. In both humans and experimental murine models, deficiencies in type-II responses is linked with enhanced control of fungal burden and diminished eosinophilia, inflammation, airway damage, and dissemination (63, 64).

Interestingly, patients with HIV infection will gradually shift from a type-I to a type-II immune response profile, thus developing an increased vulnerability to cryptococcal infection (65). Profiling studies performed by Jarvis et al. on the cytokines and chemokines produced by stimulated peripheral blood mononuclear cells (PBMCs)-derived CD4+ T-cell as well as within cerebral spinal fluid (CSF) of patients with HIV-related CM have provided some immunological associations with survival (66, 67). Increased levels of IL-6, IL-8, IL-10, IL-17, IFN-γ, tumor necrosis factor (TNF), and CCL5 within the CSF correlated with high white cell counts, macrophage activation, reduced cryptococcal burden, and survival. A high proportion of IFN-γ and TNF double producing PBMC-derived CD4+ T-cells was also associated with survival (68). This study corroborates the critical importance of maintaining a Th1/Th17 profile in both cryptococcal pulmonary infection and meningoencephalitis. Most CM studies have been performed in the context of HIV patients and *C. neoformans*; however, little is known about the immune profile in patients with non-HIV CM, particularly those with *C. gattii* infection.

## Humoral Immunity to *Cryptococcus*

As previously mentioned, serum antibodies to *Cryptococcus* can be detected in early life. However, immunocompromised patients at risk for cryptococcal infection appear to have a defect in antibody responses, such as loss of glucuronoxylomannan (GXM), a capsular component reactive B-cells, as well as overall lower levels of peripheral blood memory IgM B cells (69, 70). Lower serum GXM-IgM antibody levels in both HIV+ as well as HIV− solid organ transplant patients is also associated with increased risk for development of cryptococcosis (71–73). Antibody-mediated phagocytosis may be important as the increase in capsular size has been shown to reduce complement-mediated phagocytosis (74). Furthermore, murine studies have demonstrated that the murine equivalent of IgM memory B cells, B-1 cells, can dampen fungal growth *in vitro* and *in vivo*, by inducing an earlier T-cell response, reducing dissemination, and enhancing macrophage phagocytosis (75–78). Additionally, adoptive transfer of IgM-sufficient wild-type mouse serum into Rag1^−/−^ mice demonstrated enhanced alveolar macrophage phagocytosis and a reduction in early dissemination compared to mice treated with IgM-deficient serum. The use of vaccines or antibody therapy to boost antifungal titers may thus provide protection against the development of cryptococcal disease (79–81).

## Cryptococcal Elimination

The primary mechanism for pulmonary clearance is the formation and resolution of granulomas by macrophages. However, as previously mentioned, *Cryptococcus* is capable of surviving within resident alveolar macrophages, thus the macrophages required for clearance must be recruited and activated by CD4+ T-cell signals. Macrophages stimulated under Th1/Th17 or Th2 cytokine profiles become skewed toward either classical or alternative activation, respectively. Classically activated (M1) macrophages are primarily induced by IFNγ and lipopolysaccharide, while type-2 cytokines including IL-4 and IL-13 induce alternatively activated (M2) macrophages and function through production of proline and polyamines (82). M1 and M2 macrophages *in vitro* have demonstrated different outcomes during intracellular parasitism by *C. neoformans* with type-1/type-17 conditions having enhanced fungicidal activity (Figure 1.5) (83). Furthermore, STAT1-deficient mice, which are deficient in M1 macrophages due to an inability to generate a strong Th1 profile, have a defect in anti-cryptococcal activity, which correlated with a decrease in NO production (84). In the previously mentioned cohort of non-HIV patients with CM, although there were intact Th1 signaling found in the CSF, autopsy results revealed an overrepresentation of M2 macrophages within central nervous system (CNS) tissues (85). Similarly, patients with granulocyte-macrophage colony stimulating factor (GM-CSF) autoantibodies are also at risk for CM and have an abundance of Th1 CD4+ T-cells, but also have a skewed M2 macrophage phenotype (86, 87). Activated M1 macrophages, with CD4+ T-cells, resolve the infection by entrapping and degrading the cryptococcal propagules through the formation of granulomas (Figure 1.6).

## Brain Dissemination

Uncontrolled cryptococcal infection will inevitably disseminate into the (CNS) leading to a life-threatening CM. There are currently three known methods of cryptococcal dissemination from the lung: (1) the disruption of blood vesicle integrity allowing passive transport into the blood stream, (2) intact endothelial cells may phagocytose the spores from the lung and expulse them into the blood stream, (3) macrophages may act as a Trojan Horse by transporting phagocytosed spores to the brain, and regurgitating the spores in a process known as vomocytosis. Both microbial and host factors have been identified to be involved in CNS invasion, including cryptococcal matrix metalloprotease, production of a urease enzyme (88), and increases in host brain inositol levels (89, 90).

## Non-HIV Factors of Susceptibility

Over 1,000 cases of CM are reported to occur in previously healthy people in the U.S. annually. Studying this population reveals unique vulnerability risks, including previously undiagnosed, rare immune-associated monogenic disorders or autoimmune diseases. Patients with autoantibodies to (GM-CSF) and interferon-gamma (IFNγ) were recently demonstrated to be susceptible to CM, emphasizing the T-cell/monocyte signaling pathway that is required for a successful immune response (86, 87, 91). Interestingly, poor macrophage function was also demonstrated in a cohort of clinically refractory patients by a lack of iNOS expression and intact M2-related CD200R1 expression using immunohistochemistry of infected brain tissue (85). Further studies also demonstrated defective CSF activated macrophage TNF-α secretion, which may explain a lack of symptomatology and diagnostic delays in non-HIV related CM.

Historically, the most common syndrome associated with risk for CM is an idiopathic CD4 lymphopenia (ICL) that presents as a non-HIV-associated reduction or loss of CD4+ T-cells. The tremendous impact of CM on AIDS patients makes the importance of CD4 T-cells self-evident. However, ICL is a very heterogeneous disorder that has been implicated as a serious risk factor (92, 93) but many patients with ICL remain healthy. Recently, the concept of a “two hit” hypothesis was advanced by the finding of two ICL patients with CM who had additional autoantibodies to GMCSF or an otherwise benign, but functionally significant mutation in the IKBKG/NEMO gene, with reductions in NFKB T-cell signaling (94). Similarly, patients with monocytopenia, such as patients with a GATA2 deficiency, also have increased risk of developing CM (95–97). Monogenic disorders such as X-linked CD40L deficiency, chronic granulomatous disease, and Job syndrome are also associated with susceptibility to CM (98–100). T-cell suppressing biological therapy such as natalizumab or fingolimod is also a risk factor (101, 102).

## Cryptococcal Disease: A Reflection of Host and Microbiological Factors

Recently, there has been a greater appreciation that host damage can occur from either the toxic products of an overwhelming microbial infection or a pathological inflammatory response to the invading pathogen (Figure 1.7 and Figure 1.8), recently termed the damage-framework response (103). Cryptococcal disease is a classic example of this phenomenon, often occurring within the same patient during different stages of treatment (104, 105). For example, in the setting of HIV infection, clinical outcomes of primary therapy are related to clearance of the fungus (106, 107). However, a paradoxical immune reconstitution syndrome can also be seen in these same patients whereby, in the setting of microbiological control, reconstitution of the immune system after initiation of antiretroviral therapy (ART) results in a pathological central nervous system inflammatory response (7, 108–110). HIV-related CM further exemplifies differences in disease at these polar extremes of immune response—a recent study of 90 HIV patients with cryptococcal disease found that high levels of Th1-related cytokines INF-gamma and IL-6 were predictive of 2-week initial survival when pathogen load was high; whereas, the development of symptomatic cIRIS was associated with elevated activation with increased macrophage-related cytokines such as CCL2/MCP-1, CCL3/MIP1a, and GM-CSF (67).

Similar to HIV-related disease, in the initial stages of therapy of non-HIV patients, failure to achieve negative CSF fungal cultures at 2 weeks is associated with clinical failure (107). However, many more such patients develop refractory symptoms and/or clinical deterioration despite microbiological control, recently described in previously healthy patients as a postinfectious inflammatory response syndrome. Similar to that encountered with cIRIS in HIV, these patients have an activated CD4+ T-cell intrathecal compartment with minimal Th2 presence (85). Additionally, these patients have high levels of CD4+ T-cells in the CSF and within the intracranial Virchow–Robin channels, which displayed an activated phenotype, as measured by HLA-DR4 and CD56 positivity. Elevated Th1 CSF soluble cytokines such as INF-gamma and interferon-related CXCL10 confirmed the activated T-cell phenotype. In addition, a strong relationship between the T-cell activation marker, sCD27, and elevations of the axonal damage marker, neurofilament light chain protein, suggest that such inflammation is not a benign event, but pathological (85, 111). These observations were surprising as these conditions typically define a successful immune response, as understood from susceptibility studies in HIV-related disease. However, as described above, many of the previously healthy have defects in macrophage polarization, allowing disease susceptibility in the face of unrestrained T-cell-mediated host damage. Such findings also highlight the limitations of applying disease principles from one host to another without careful consideration.

## Treatment: Toward a More Rational Approach to Adjunctive Therapy

Treatment strategies for patients with CM should be developed to address damage caused by both the microbe and the pathological immune response; the disease framework is a useful guide (Figure 1.7 and Figure 1.8) (104). In the initial therapy of all patients, treatment with antifungals with a fungicidal agent such as amphotericin B is paramount and is typically continued with oral azole therapy to prevent relapse after initial negative CSF cultures (112–114). Those with low immunity (HIV prior to ART or patients with a skewed Th2 response to *Cryptococcus*), may benefit from potential immune adjuvants such as IFNγ to accelerate microbiological control (115). In these cases, rates of clearance of CSF fungal cultures (early fungicidal activity) may be an important parameter (67, 106, 107). However, in HIV patients who have developed cryptococcal immune reconstitution inflammatory syndrome (cIRIS) or in refractory non-HIV patients after microbiological control, attention needs to be drawn to the host damage side of the disease model—to minimize pathological effects of a dysregulated host response. In this setting, application of immune enhancers such as IFNγ therapy (116) may lead to exacerbated inflammation and potentially cause irreparable neurological damage (85). In these patients, use of adjunctive immunosuppression including corticosteroids is increasingly reported to suppress pathological inflammatory responses and control cerebral edema, improving clinical response (85, 115–120). In the previously healthy CM patient, for example, successful application of adjunctive corticosteroids in refractory disease or after clinical deterioration requires a personalized strategy. When resources are available, CSF culture negativity and measures of CSF inflammation such as sCD27, CSF glucose, or choroid plexitis or ependymitis by MRI imaging can provide specific biomarkers of the relative contributions of microbe and host toward understanding an individual patient’s condition (85, 120, 121). Biomarkers may also be useful guides during corticosteroid tapers to prevent exacerbations.

However, applying this type of therapy successfully requires careful attention to concurrent microbial control, as corticosteroids suppress innate and acquired immune responses needed to maintain fungal clearance (122, 123). Indeed, corticosteroids can have a deleterious effect when applied without pre-established microbial control during primary therapy of HIV-related CM (Figure 1.7) (124). The complex heterogeneity of clinical pathologies that occur in various patients during HIV-related CM requires a thoughtful approach that considers the evolving damage caused by both the fungus and the immune response within defined sub-groups. In resource-limited regions, more studies are needed to understand the damage-response framework as it relates to CM in poorly controlled HIV infection, and to identify markers that can tailor resource appropriate therapies. Strategies to prevent CM in HIV (125) and to diagnose or empirically treat co-infections (126) may have tremendous impact on outcomes given the high prevalence of disease in some geographic regions (8). Risks for co-infections, such as tuberculosis and bacterial infections can be exacerbated without specific therapy in the presence of corticosteroids (127).

Other populations with risks for CM such as solid organ transplant recipients also require tailored approaches to prevention and treatment. In populations other than HIV, the feasibility of prevention strategies is limited due to a lower prevalence of disease. Much more attention is needed to better define appropriate therapeutic strategies. Since diagnosis typically occurs late, fungal burden can be high at diagnosis; at the same time, relatively intact and variable inflammatory responses can lead to exuberant inflammatory neurological damage similar to cIRIS (128). In these patients, attention needs to be focused on personalizing therapies according to which side of the host damage framework is most responsible for neurological pathology (128, 129).

## Author Contributions

The bulk of this manuscript was written by WE under the guidance of PW. PW and KM provided revisional comments and suggestions on both content and organization of this manuscript.

## Conflict of Interest Statement

The authors declare that the research was conducted in the absence of any commercial or financial relationships that could be construed as a potential conflict of interest.
